# *Mycobacterium leprae* on Palatine Tonsils and Adenoids of Asymptomatic Patients, Brazil

**DOI:** 10.3201/eid2610.191267

**Published:** 2020-10

**Authors:** Marilda Aparecida Milanez Morgado de Abreu, Gisele Alborghetti Nai, Juliana D’Andrea Molina, Rafael Tomaz Gomes, Natalia de Paula, Ana Maria Roselino

**Affiliations:** Regional Hospital, Presidente Prudente, Brazil (M.A.M. Morgado de Abreu, R.T. Gomes);; University of Oeste Paulista, Presidente Prudente (M.A.M. Morgado de Abreu, G.A. Nai, J.A. Molina);; and University of São Paulo, Ribeirão Preto, Brazil (N. de Paula, A.M. Roselino).

**Keywords:** adenoids, immunohistochemistry, infection, leprosy, *Mycobacterium leprae*, palatine tonsils, PCR, Brazil, tuberculosis and other mycobacteria, bacteria

## Abstract

We investigated palatine tonsil and adenoid specimens excised from otorhinolaryngological patients in a leprosy-endemic region of Brazil. Fite-Faraco staining identified *Mycobacterium* spp. in 9 of 397 specimen blocks. Immunohistochemistry and molecular analysis confirmed the presence of *Mycobacterium leprae,* indicating that these organs can house *M. leprae* in persons inhabiting a leprosy-endemic region.

Leprosy is a chronic infectious disease caused by *Mycobacterium leprae* that especially affects skin and peripheral nerves (*1*). In 2018, the registered global prevalence in the 6 World Health Organization regions was 184,238 cases (0.24/10,000 population), showing a decrease of 8,475 cases over the previous year (*2*). Although its incidence in Brazil has declined during 2009–2018, leprosy continues to be a major public health problem at the national level (*1*). Reports of *M. leprae* resistance against antimicrobial drugs used in multidrug therapy raise concern about the future of leprosy treatment (*2*). Therefore, not only does leprosy persist, but the emergence of multidrug-resistant *M. leprae* is a potential threat to global public health (*3*,*4*).

Although the exact mode of leprosy transmission is not known, it is thought that the upper respiratory tract, in particular the nasal mucosa, is the usual site of primary infection (*3*). Because we have previously identified *M. leprae* in oral mucosa of leprosy patients (*5*), we aimed to investigate other anatomic sites that could host this microorganism to clarify the epidemiology and transmission mechanisms of leprosy.

In this study, we hypothesized that *M. leprae*, after penetration through the airway mucosa, could infect the palatine tonsils and adenoids, because these organs represent the first immune defense line against inhaled or ingested antigens (*6*). We also theorized that if leprosy is a highly contagious disease (*1*), a considerable part of the population in endemic regions might be infected with *M. leprae*.

We conducted a cross-sectional study of 397 paraffin-embedded blocks of palatine tonsils and adenoids extracted from 144 patients due to otorhinolaryngological indication during 2011–2016 at the Regional Hospital, Presidente Prudente, Brazil. The local Research Ethics Committee approved the study (protocol #1.920.994).

Microscopic analysis using hematoxylin-eosin staining did not reveal granulomas. We analyzed 50 fields in the 100× objective (1,000× magnification) per slide stained with Fite-Faraco; of the positive cases (9 [2.3%] slides from 8 [5.6%] patients, 6 men and 2 women [mean age 11 + 5.5 years]), we observed only 1 acid-fast rod per slide. We studied all the blocks of these 8 patients, a total of 20 blocks (Table).

**Table:** Patient data and results of Fite-Faraco staining, immunohistochemistry with anti**–**PGL-I antibody, and PCR assays in study of *Mycobacterium* *leprae* on palatine tonsils and adenoids, Brazil, 2019*

Patient no.	Age, y/sex	Lymphoid organ	Fite-Faraco stain	IHC anti–PGL-I	PCR RLEP	*M. leprae* identification by DNA sequencing, %
1	20/F	AD	+	+	+	ND
2	19/M	RPT	+	+	+	ND
		LPT	–	+	+	ND
3	10/M	RPT	+	+	+	100
		LPT	–	+	+	ND
4	9/M	RPT	–	+	+	ND
		LPT	+	+	+	99
		AD	+	+	+	ND
5	7/M	RPT	–	+	+	ND
		LPT	+	+	+	ND
		AD	–	+	+	ND
6	4/M	RPT	–	+	+	ND
		LPT	+	+	+	99
		AD	–	–	+	ND
7	13/F	RPT	+	+	+	98
		LPT	–	+	+	ND
		AD	–	+	+	ND
8	6/M	RPT	–	–	–	ND
		LPT	+	+	+	ND
		AD	–	+	+	98
Total positive results			9	18	19	

*AD, adenoid; PGL-I, phenolic glycolipid I; IHC, immunohistochemistry; LPT, left palatine tonsil; ND, not determined; RLEP, *M. leprae* repetitive DNA sequence; RPT, right palatine tonsil; –, negative; +, positive.

Immunohistochemistry with 1:20,000 anti–phenolic glycolipid-I (anti-PGL-I) antibody (Bei Resources, https://www.beiresources.org), specific for *M. leprae*, was conducted with the Mach 1 polymer-based biotin-free detection kit (Biocare Medical, https://biocare.net) (*5*). We used deparaffinized skin sections from multibacillary leprosy as positive control. For the negative control, we omitted the antibody. To confirm specificity, we used deparaffinized skin sections from paucibacillary leprosy and atopic dermatitis (excluding inflammatory cell recognition by the antibody), normal human scalp, and tuberculous lung section.

We extracted DNA from paraffin sections with isopropanol-ammonium acetate (*1*). The resulting DNA was used in conventional PCR with sense 5¢-ATTTCTGCCGCTGGTATCGGT-3¢ and antisense 5¢-TGCGCTAGAAGGTTGCCGTAT-3¢ primers (ThermoFisher Scientific, https://www.thermofisher.com) to amplify *M. leprae* microsatellite sequences, according to a previous report (*7*). We assessed amplicons of 148 bp on 2% agarose gel. The assays included negative (DNA omission) and positive (DNA from multibacillary leprosy skin sample) controls. We confirmed specificity with DNA extracted from *M. tuberculosis* culture. In addition, we conducted PCR with TB1/TB2 primers to detect *Mycobacterium* spp. and with T4/T5 primers to detect *M. tuberculosis* (*5*).

Immunohistochemistry was positive in 18/20 (90%) blocks. By PCR, 19 (95%) were positive with RLEP and 5 were simultaneously positive with TB1/TB2; all 19 positive by PCR were negative by T4/T5 primers. Samples that tested negative in IHC or PCR were also negative in Fite-Faraco staining (Table).

Little attention has been paid to the role that mucosa-associated lymphoid tissue (MALT) plays in the mechanisms used by mycobacteria during host invasion. In tuberculosis infection, bacilli cross mucous membranes and penetrate into palatine tonsils and adenoids, where they initiate an immune response. However, bacilli may develop immune-evasion strategies and disseminate into the organism or return to the mucosa surface and be eliminated to the environment (*8*). This process might also occur in leprosy, but to our knowledge there are no reports on this subject.

Our results corroborate the hypothesis that *M*. *leprae* bacilli infect palatine tonsils and adenoids. Prospective studies with a larger population group are necessary to clarify these findings. We could not infer from this retrospective study with paraffinized samples whether patients who had positive results for *M. leprae* identification had leprosy or were asymptomatic carriers. In both clinical scenarios, however, our findings indicate that palatine tonsils and adenoids may represent reservoirs for *M. leprae* bacilli in persons inhabiting a leprosy-endemic region. 
